# Association of some *Campylobacter jejuni* with *Pseudomonas aeruginosa* biofilms increases attachment under conditions mimicking those in the environment

**DOI:** 10.1371/journal.pone.0215275

**Published:** 2019-04-10

**Authors:** Amy Huei Teen Teh, Sui Mae Lee, Gary A. Dykes

**Affiliations:** 1 School of Science, Monash University, Jalan Lagoon Selatan, Bandar Sunway, Selangor Darul Ehsan, Malaysia; 2 School of Public Health, Curtin University, Bentley, Western Australia, Australia; United States Department of Agriculture, Agricultural Research Service, UNITED STATES

## Abstract

*Campylobacter jejuni* is a microaerophilic bacterial species which is a major food-borne pathogen worldwide. Attachment and biofilm formation have been suggested to contribute to the survival of this fastidious bacteria in the environment. In this study the attachment of three *C*. *jejuni* strains (*C*. *jejuni* strains 2868 and 2871 isolated from poultry and ATCC 33291) to different abiotic surfaces (stainless steel, glass and polystyrene) alone or with *Pseudomonas aeruginosa* biofilms on them, in air at 25°C and under static or flow conditions, were investigated using a modified Robbins Device. Bacteria were enumerated and scanning electron microscopy was carried out. The results indicated that both *C*. *jejuni* strains isolated from poultry attached better to *Pseudomonas aeruginosa* biofilms on abiotic surfaces than to the surfaces alone under the different conditions tested. This suggests that biofilms of other bacterial species may passively protect *C*. *jejuni* against shear forces and potentially oxygen stress which then contribute to their persistence in environments which are detrimental to them. By contrast the *C*. *jejuni* ATCC 33291 strain did not attach differentially to *P*. *aeruginosa* biofilms, suggesting that different *C*. *jejuni* strains may have alternative strategies for persistence in the environment. This study supports the hypothesis that *C*. *jejuni* do not form biofilms *per se* under conditions they encounter in the environment but simply attach to surfaces or biofilms of other species.

## Introduction

*Campylobacter jejuni* is a Gram-negative, curved or spiral rod-shaped and motile microaerophilic bacterial species. It is one of the most common causes of bacterial gastrointestinal food-borne infection worldwide [1]. Poultry is regarded as the primary source of human *C*. *jejuni* infections, with approximate 80% of retail-ready chicken products reported to be contaminated with it [2, 3]. The most common route of human *C*. *jejuni* infections is suggested to be through consumption of undercooked poultry and raw milk [4–6]. In addition, handling of contaminated poultry may also contribute to cross-contamination of other foods and lead to indirect *C*. *jejuni* infections [7].

*C*. *jejuni* is fastidious, generally requiring specific atmospheres and temperatures to grow [8]. It is also susceptible to different environmental and food processing induced stressors, including oxygen, temperature and pH stress [9, 10]. Despite its sensitivity to these stressors, *C*. *jejuni* is commonly isolated from the environment [9, 11]. *C*. *jejuni* has been isolated from biofilms in watering supplies and plumbing systems of animal processing plants and animal husbandry facilities [6, 12]. The ability of *C*. *jejuni* to form biofilm has been suggested to protect them from the stressful conditions and contribute to their survival in the environment [12–15].

In nature biofilms consist of mono- or mixed-species populations, with most bacterial biofilms in the environment made up of a mixture of different species [16]. *C*. *jejuni* has been previously reported to be a poor biofilm initiator, and the presence of mono-species *C*. *jejuni* biofilms have largely been reported under controlled conditions that support its growth [14, 17, 18]. The possibility of *C*. *jejuni* being the primary colonizer for biofilm formation in the environment, including in food processing (such as poultry) plants, is therefore low [18]. Several studies have reported that *C*. *jejuni* is able to attach to, and form mixed-species biofilms with, other bacterial species such as *Pseudomonas aeruginosa*, *Escherichia coli*, *Enterococcus faecalis* and *Staphylococcus simulans* [15, 17, 19, 20], which are commonly found in poultry environments. Work on *C*. *jejuni* biofilms mostly entails mono-species studies performed under static incubation conditions with little or no shear force applied. These studies have also tended to have been performed in microaerobic atmospheres at temperatures above 30°C, suitable for the growth of these pathogens. These conditions are different from the conditions they are likely to face in the environment and therefore do not provide sufficient insight how *C*. *jejuni* might behave in the environment. It has also been suggested that *C*. *jejuni* are unable to form biofilms *per se* in the environment. Rather they may simply attach to and interact with surfaces, or preformed biofilms of other species, particularly at temperatures that do not permit them to grow [18]

In this study the attachment of *C*. *jejuni* to abiotic surfaces or to *P*. *aeruginosa* biofilms, under flow and static conditions in air at 25°C was investigated. *P*. *aeruginosa* was used in this study as this bacterial species is found in environments associated with poultry processing and *P*. *aeruginosa* has been reported to coexist with *C*. *jejuni* [20, 21]. At the temperature used *P*. *aeruginosa* was able to grow but *C*. *jejuni* could not as it does not grow at temperatures below 30°C [22].

## Materials and methods

### Bacterial strains and growth conditions

Two *C*. *jejuni* strains (2868 and 2871) isolated independently from poultry obtained from retail outlets in Malaysia [23] were used in this study. Whole genomes of the strains have been sequenced [24]. *Campylobacter jejuni* ATCC 33291 and *Pseudomonas aeruginosa* ATCC 27853 obtained from the American Type Culture Collection (Manassas, USA) were also used in this study. All the *C*. *jejuni* strains were maintained at -80°C in nutrient broth no. 2 (NB2, Oxoid, UK) and 15% glycerol and were resuscitated on *Campylobacter* blood-free selective agar base (Oxoid, UK) with incubation at 37°C for 48 h under microaerobic conditions (5% O_2_, 10% CO_2_ and 85% N_2_) generated using Campygen (Oxoid, UK) in an anaerobic jar (Oxoid, UK). *P*. *aeruginosa* was maintained at -80°C in Luria-Bertani broth (LB, Oxoid, UK) and 15% glycerol and was resuscitated on LB agar (Oxoid, UK) with incubation at 37°C for 24 h under aerobic conditions.

### Assessment of biofilm formation

A modified Robbins device (MRD, LPMR-12PSF; Tyler Research Co-operation, Canada) was used to assess the ability of the *C*. *jejuni* strains to attach to surfaces or to biofilms of *P*. *aeruginosa* under static and flow conditions at 25°C in Mueller-Hinton broth (MHB, Oxoid, UK). Three types of biostuds (50mm^2^), stainless steel, glass and polystyrene (Tyler Research Co-operation, Canada), were used in the biofilm formation assay. Before being used in the biofilm assay, the MRD along with stainless steel or glass biostuds were sterilized by autoclaving. Polystyrene biostuds were sterilized by soaking in 70% ethanol and treated under UV for 30 min before assembly into the sterilized MRD using aseptic technique. The MRD was placed in a biosafety cabinet at ambient room temperature under normal aerobic conditions throughout the incubation period.

#### Preparation of bacterial cultures

The *C*. *jejuni* strains were grown as sessile cultures on *Campylobacter* blood-free selective agar under microaerobic conditions for 48 h at 37°C. Growth of the inoculum under these conditions was previously shown to result in better subsequent attachment and biofilm formation as compared to a range of other growth conditions examined [25]. After incubation colonies on the agar plates were harvested by suspending them in MHB and adjusting the cell concentration to 10^7^ CFU ml^-1^. A 15 ml aliquot of the cell suspension was taken and used for the attachment assay. *P*. *aeruginosa* was grown in MHB for overnight at 37°C. The optical density (OD) of the overnight culture was then adjusted to a cell concentration of 10^7^ CFU ml^-1^ and 15 ml of the cell suspension was used for the biofilm assay.

#### Biofilm growth under static and flow conditions

Attachment was investigated initially under static conditions. A miniature peristaltic pump (BQ50-1J, PLT Scientific, Malaysia) was used to pump the culture through the MRD at a rate of 1 ml min^-1^. For the mono-species experiment 15 ml of a *C*. *jejuni* cell suspension was pumped into the MRD. For the mixed-species experiment in which *C*. *jejuni* was present during *P*. *aeruginosa* biofilm initiation, 15 ml of *C*. *jejuni* cell suspension was mixed with 15 ml of *P*. *aeruginosa* cell suspension before 15 ml of the mixed-species culture was pumped into the MRD. After the MRD was filled with the bacterial culture(s) the flow was stopped and the cells were allowed to attach for 2h at 25°C. The bacterial suspension was then replaced with 15 ml of fresh MHB for 15 min. The flow was stopped and the MRD was incubated for 24 h at 25°Cu nder static conditions to allow biofilm formation and attachment. To investigate *C*. *jejuni* attachment to pre-formed *P*. *aeruginosa* biofilms, 15 ml of *P*. *aeruginosa* cell suspension was pumped into the MRD and incubated for 24 h before 15 ml of *C*. *jejuni* cell suspension was pumped into the MRD. After 2 h of incubation, 15 ml of fresh MHB was pumped into the MRD which was then incubated for a further 24 h under static conditions. Attachment was subsequently investigated under flow conditions. The same set-up procedure as described for the static conditions above was used except after 2 h of incubation fresh MHB was pumped through the MRD continuously for 24 h at a rate of 1 ml min^-1^.

#### Quantification of attachment

After incubation each biostud was removed and rinsed with phosphate buffer saline (PBS; 1^st^ BASE, Singapore) before being placed in a 15 ml centrifuge tube containing 1 ml of PBS. Each tube was then sonicated at room temperature at a frequency of 35 kHz for 10 min using a water bath soniator (LC-130H; ELMA, Germany). The tube was then vortexed for 1 min before serial diluted in PBS and plated on modified cefoperazone charcoal deoxylate agar (mCCDA; Oxoid, UK). The plates were incubated at 42°C for 48 h before enumeration. Growth of *P*. *aeruginosa* is inhibited on mCCDA.

### Scanning electron microscopy (SEM)

Biofilms were examined on glass biostuds using SEM as previously described [26] with some modifications. Briefly, the biostuds on which mono-species or mixed-species had developed under static and flow conditions were washed in PBS and then fixed with 2.5% (vol/vol) glutaraldehyde (Sigma Aldrich, USA) in PBS for 2 hours. The fixed discs were washed in PBS followed by dehydration in a graded alcohol series (30, 50, 70, 80, 90, 95 and 100% ethanol) before storing in a desiccator (Kartell, Italy) overnight. The discs were then gold-sputtered using a sputter coater (Q150RS; Quorum, UK) and viewed under a scanning electron microscope (S-3400N; Hitachi, Japan).

### Statistical analysis

All experiments were performed in triplicates with independently grown cultures. All the statistical analysis was performed using SPSS 18 software (PASW Statistics 18; SPSS Inc.). A multi-factor analysis of variance (ANOVA) was used to compare attachment by the different *C*. *jejuni* strains under different conditions and pairwise comparisons of the means were conducted using Tukey’s post hoc test at a 95% confidence level. A one-way ANOVA was also carried out to compare the number of *C*. *jejuni* cells recovered in the three attachment experiments for each of the bacterial strains under both static or flow conditions.

## Results

### Attachment of different *C*. *jejuni* strains

**Fig 1:**
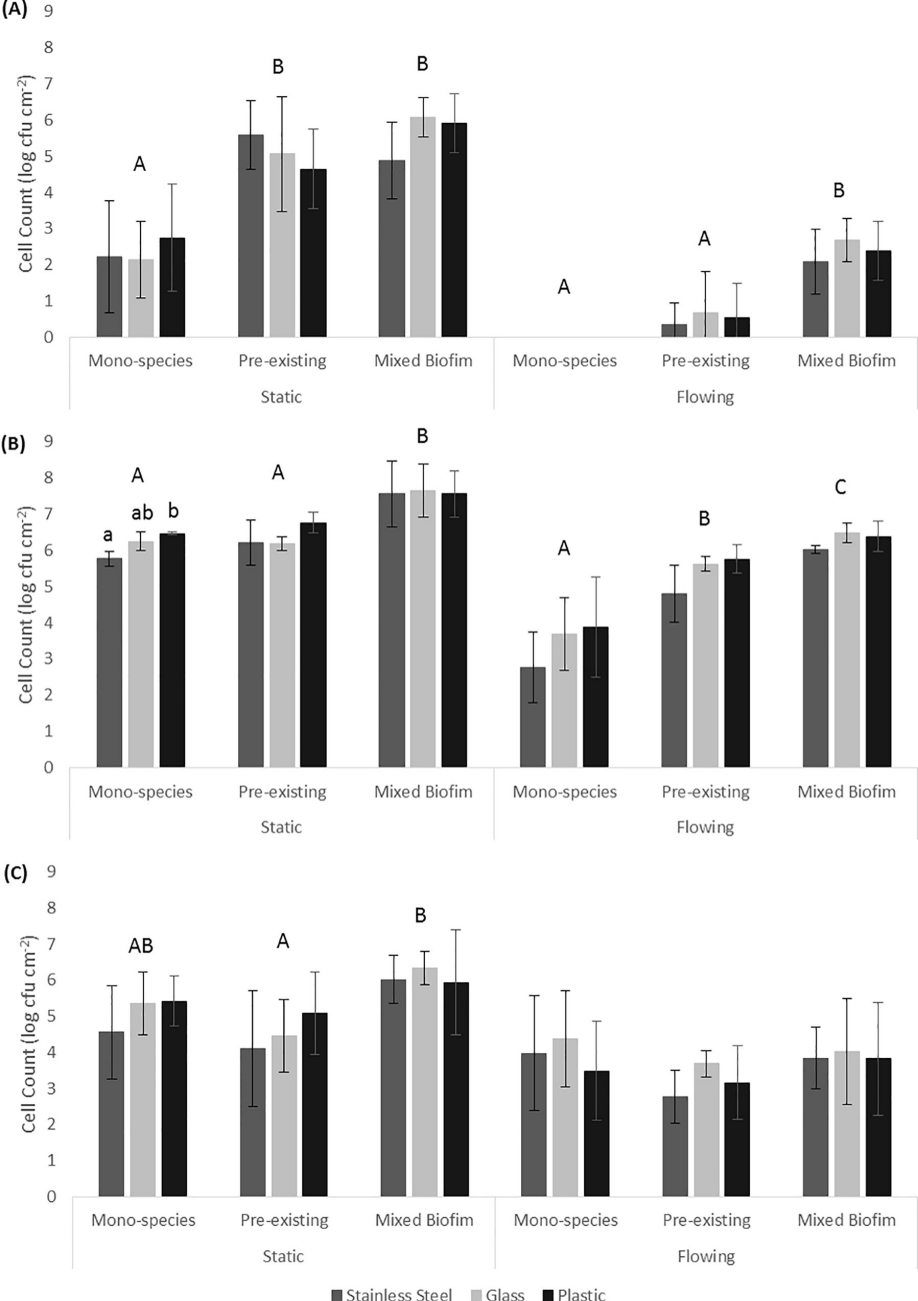
**Cell counts of *C*. *jejuni* (A) strain 2868, (B) strain 2871 and (C) strain ATCC 33291 attached to different abiotic surfaces (stainless steel, glass or plastic) alone or in association with *P*. *aeruginosa* under static or flow conditions.** All results are presented in mean ± SD where n = 3. Different uppercase letters represent significant difference (p<0.05) between the cell count of the bacteria under different conditions within static or flow conditions. Different lowercase letters represent significant difference (p<0.05) between the cell count of the bacteria on different surfaces.

Numbers of *C*. *jejuni* attaching in the presence or absence of *P*. *aeruginosa* under static or flow conditions on three different abiotic surfaces (stainless steel, glass and polystyrene) are shown in Fig 1. A multi-factor ANOVA (S1 Table) showed that, taken together under all the conditions tested, there was a significant difference in attachment of the three strains (p<0.05; F = 149.36). Tukey’s *post hoc* test was carried out and the results showed that strain 2871 attached in the greatest numbers followed by strain ATCC 33291 and lastly strain 2868 (p<0.05).

### Attachment to different abiotic surfaces

Comparison of *C*. *jejuni* attachment to the different abiotic surfaces indicated that all three strains tested attached to the three different surfaces tested. As shown in Fig 1 there was no significant difference (p>0.05) in the number of *C*. *jejuni* cells recovered from the three different surfaces under all the different treatments, except for strain 2871 in the mono-species experiment under static conditions. Specifically strain 2871 attached in significantly higher levels (p<0.05) to plastic, which is a more hydrophobic surface, as compared to stainless steel.

### Attachment under different environmental conditions

The results shown in Fig 1 indicated that in addition to attaching to abiotic surfaces *C*. *jejuni* was able to attach to pre-existing *P*. *aeruginosa* biofilms or to *P*. *aeruginosa* biofilms during initiation under static or flow conditions. Based on the results obtained from the multi-factor ANOVA (S1 Table), the numbers of *C*. *jejuni* attaching was significantly lower (p<0.05; F = 213.726) under flow conditions as compared to static conditions. Similarly, all the strains showed a significant reduction (p<0.05) in the number of *C*. *jejuni* cells recovered under flow conditions as compared to static conditions.

**Fig 2:**
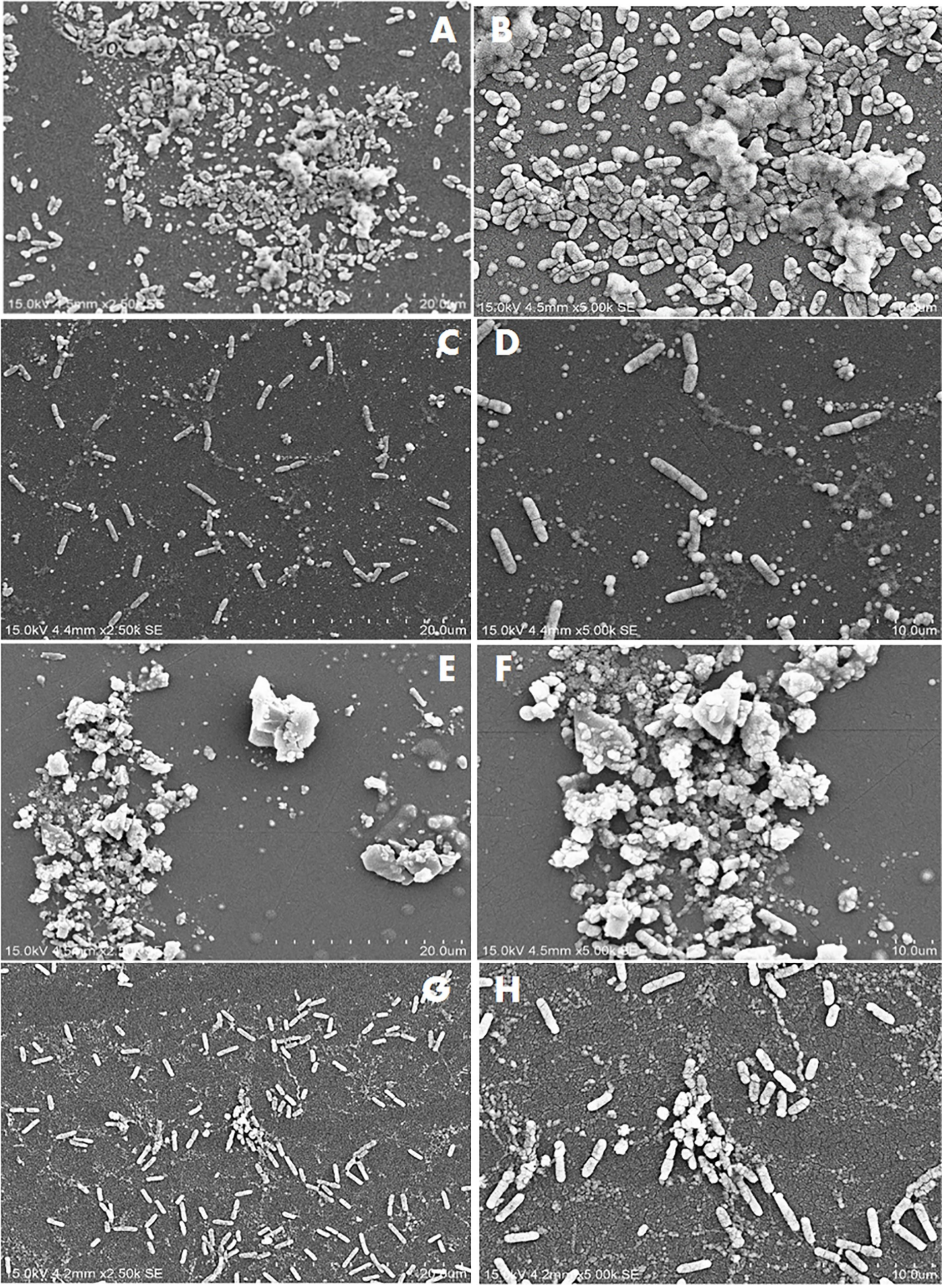
SEM images of attachment of *C. jejuni* strain 2868 to *P. aeruginosa* biofilms under different conditions. (A) and (B) Pre-existing *P*. *aeruginosa* biofilm under static conditions; (C) and (D) Pre-existing *P*. *aeruginosa* biofilm under flow conditions; (E) and (F) *P*. *aeruginosa* biofilms with *C*. *jejuni* added during initiation under static conditions; (G) and (H) *P*. *aeruginosa* biofilms with *C*. *jejuni* added during initiation under flow conditions.

**Fig 3:**
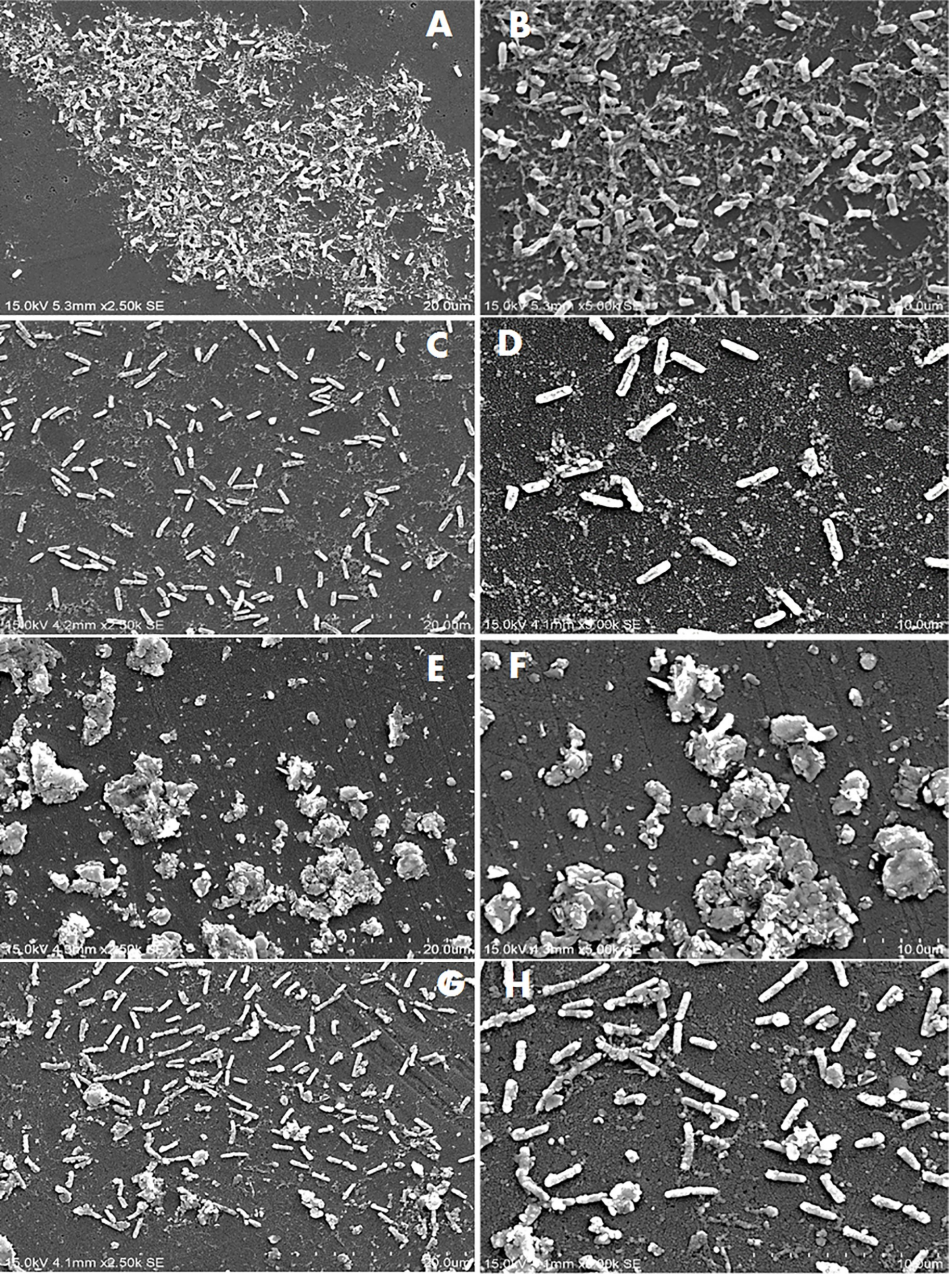
SEM images of attachment of *C*. *jejuni* strain 2871 to *P*. *aeruginosa* biofilms under different conditions. (A) and (B) Pre-existing *P*. *aeruginosa* biofilm under static conditions; (C) and (D) Pre-existing *P*. *aeruginosa* biofilm under flow conditions; (E) and (F) *P*. *aeruginosa* biofilms with *C*. *jejuni* added during initiation under static conditions; (G) and (H) *P*. *aeruginosa* biofilms with *C*. *jejuni* added during initiation under flow conditions.

**Fig 4:**
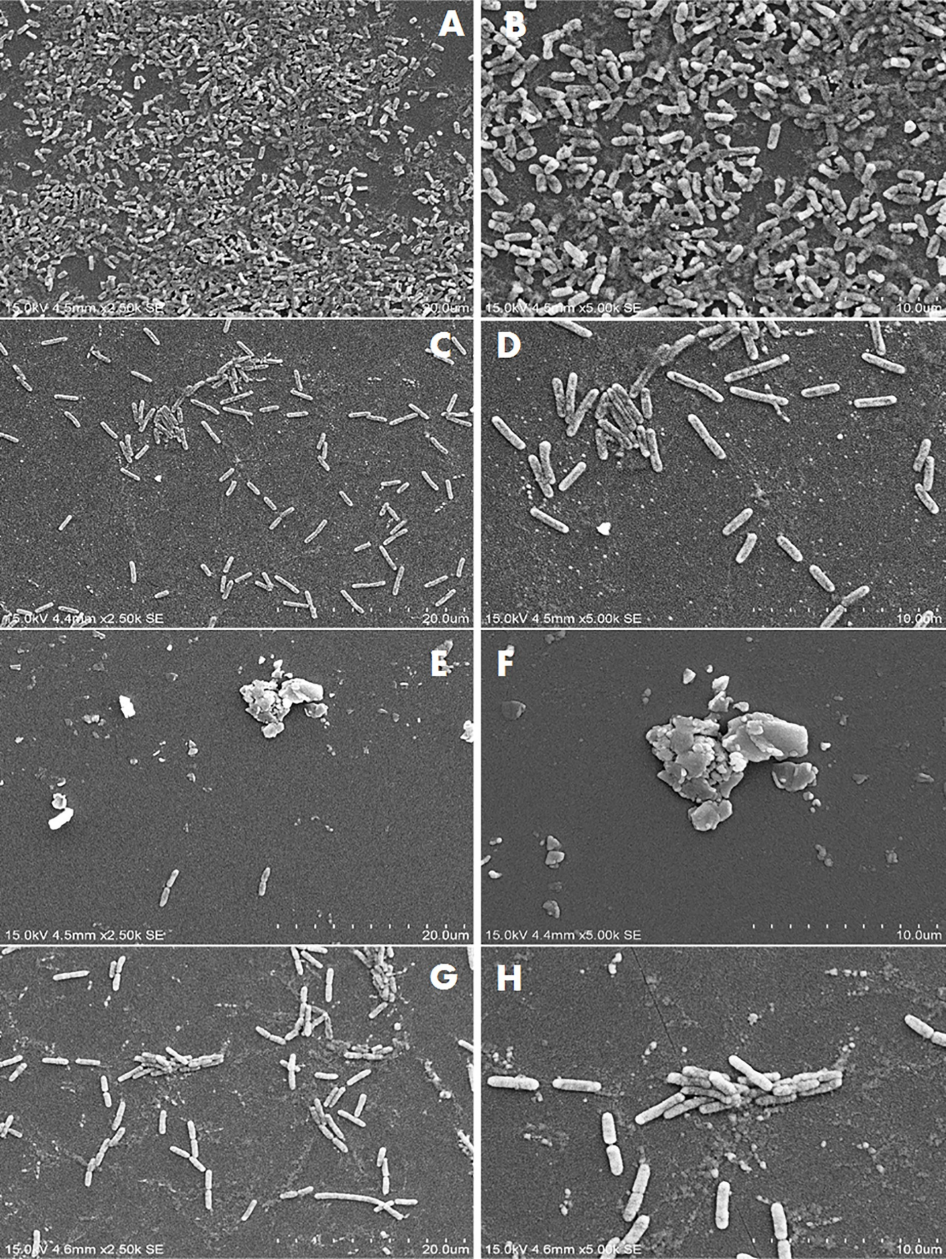
SEM images of attachment of *C*. *jejuni* strain ATCC 33291 to *P*. *aeruginosa* under different conditions. (A) and (B) Pre-existing *P*. *aeruginosa* biofilm under static conditions; (C) and (D) Pre-existing *P*. *aeruginosa* biofilm under flow conditions; (E) and (F) *P*. *aeruginosa* biofilms with *C*. *jejuni* added during initiation under static conditions; (G) and (H) *P*. *aeruginosa* biofilms with *C*. *jejuni* added during initiation under flow conditions.

**Fig 5:**
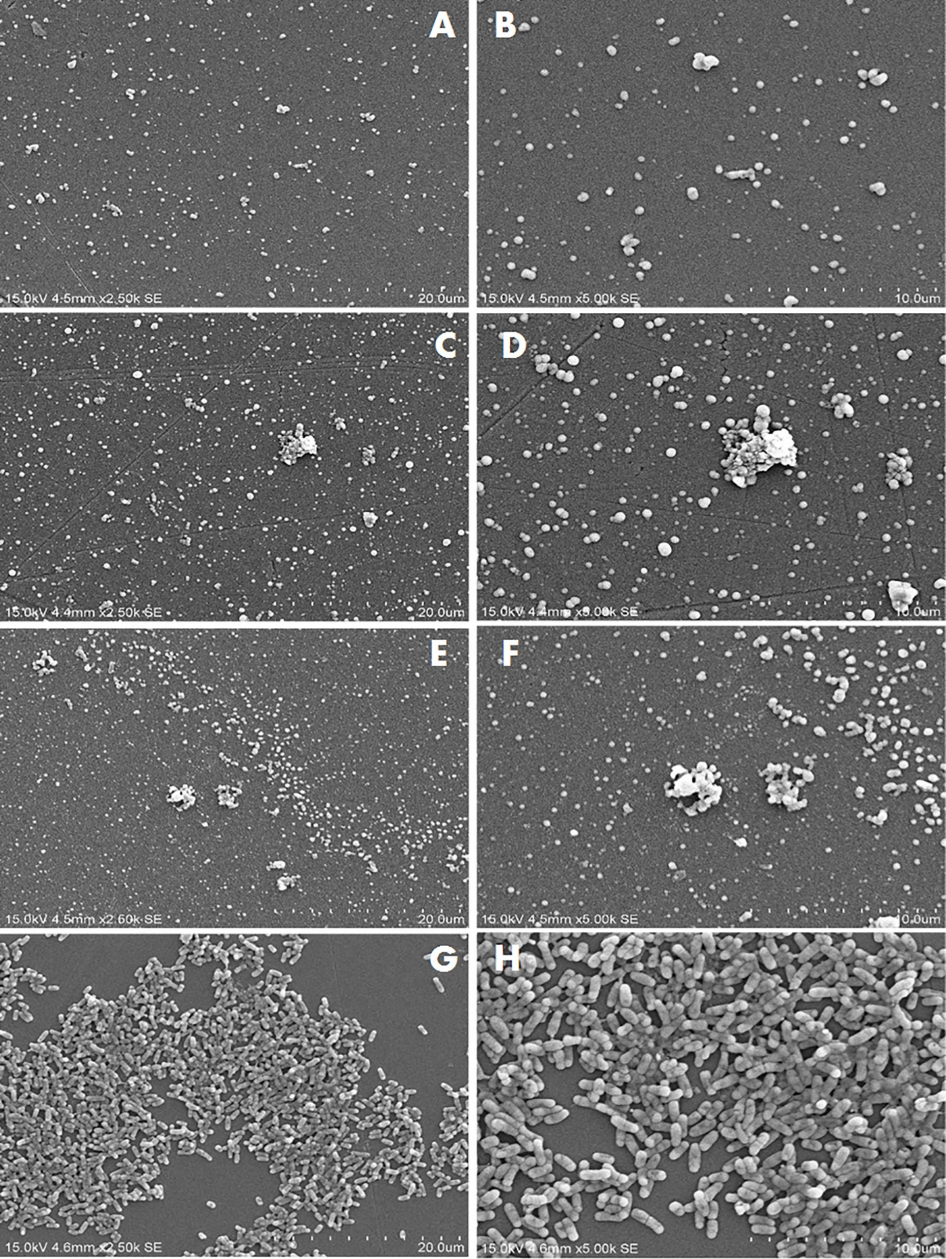
SEM images of mono-species attachment to abiotic surfaces. (A) and (B) *C*. *jejuni* strain 2865; (C) and (D) *C*. *jejuni* strain 2871; (E) and (F) *C*. *jejuni* strain ATCC 33291; (G) and (H) *P*. *aeruginosa* ATCC 27853 under static conditions.

Figs 2–4 show the SEM images of *C*. *jejuni* strain 2865, 2868 and ATCC 33291 cells, respectively, attached to pre-existing *P*. *aeruginosa* biofilm and to *P*. *aeruginosa* biofilms during initiation under static or flow conditions. SEM images for *C*. *jejuni* mono-species experiments and *P*. *aeruginosa* mono-species biofilm formation under static conditions are shown in Fig 5. SEM on *C*. *jejuni* mono-species experiments under flow conditions are not presented as the number of cells were too low to be visualised. As shown in the SEM images, *P*. *aeruginosa* biofilms with *C*. *jejuni* attached formed under static conditions (Figs 2A, 2B, 2E, 2F, 3A, B, 3E, 3F, 4A, 4B, 4E and 4F) were more compact and closely aggregated with extensive extracellular polymeric matrix (EPM) as compared to biofilms formed under flow conditions where the bacteria were loosely or not connected by EPM and appeared as planktonic bacteria (Figs 2C, 2D, 2G, 2H, 3C, 3D, 3G, 3H, 4C, 4D, 4G and 4H). On the other hand, individual or small clusters of cells of *C*. *jejuni* which appeared to be cocci in shape were observed in the mono-species experiments (Fig 5A–5F).

The multi-factor ANOVA (S1 Table) indicates there was a significant difference in the number of *C*. *jejuni* cells recovered from the different types of attachment experiments (p<0.05; F = 49.039). The highest numbers of cells were recovered from experiments in which they were attached to *P*. *aeruginosa* biofilms during initiation, followed by attachment to pre-existing *P*. *aeruginosa* biofilms and lastly from the mono-species experiments. A one-way ANOVA was then carried out to compare the number of cells recovered from different types of attachment experiments for each of the bacterial strains under static or flow conditions. As shown in Fig 1, for strain 2868 cells recovered from pre-existing *P*. *aeruginosa* biofilms or when attached to *P*. *aeruginosa* biofilms during initiation were significantly higher (p<0.05) as compared to those in the mono-species experiments. Under flow conditions no cells were recovered from mono-species experiments but a significantly higher number of cells (approximate 2 to 2.5 log) were recovered from experiments where they were attached to *P*. *aeruginosa* biofilms during initiation. For strain 2871 *C*. *jejuni* cells recovered from experiments where they were attached to *P*. *aeruginosa* biofilms during initiation were significantly higher (p<0.05) as compared to those from mono-socies experiments and pre-existing *P*. *aeruginosa* biofilms under static conditions. Under flow conditions, cell numbers recovered from both pre-existing *P*. *aeruginosa* biofilms or experiments where they were attached to *P*. *aeruginosa* biofilms during initiation were significantly higher (p<0.05) as compared to those from the mono-species experiments.

The *P*. *aeruginosa* biofilms that formed when *C*. *jejuni* was present during initiation appeared as clusters of cells surrounded with extensive EPM under static conditions (Figs 2E, 2F, 3E and 3F). Under flow conditions, although the cells were more dispersed, small clusters of cells surrounded with EPM can still be observed (Figs 2G, 2H, 3G and 3H). When *C*. *jejuni* was added to pre-existing *P*. *aeruginosa* biofilms, higher numbers of *C*. *jejuni* cells (approximately 2 log) were recovered under static conditions for strain 2868 but not for strain 2871. As can be seen in Fig 2A and 2B, when *C*. *jejuni* 2868 was added to pre-existing *P*. *aeruginosa* biofilms cells were not clustered but appeared in a single layer connected with EPM. A single layer of bacteria cells connected with flattened EPM was observed when *C*. *jejuni* 2871 was added to pre-existing *P*. *aeruginosa* biofilm (Fig 3A and 3B) and this may reduce the number of *C*. *jejuni* cells entrapped in the biofilm. For *C*. *jejuni* ATCC 33291 there was no significant difference in the number of *C*. *jejuni* cells recovered when they were added to pre-existing *P*. *aeruginosa* biofilms or experiments where they were attached to *P*. *aeruginosa* biofilms during initiation as compared to the mono-species experiments under both static and flow conditions (Fig 1). Under static conditions *C*. *jejuni* ATCC 33291 on pre-existing *P*. *aeruginosa* biofilm appeared as a single layer cells connected with flattened EPM (Fig 4A and 4B). Although clusters of cells surrounded with EPM were observed when *C*. *jejuni* ATCC 33291 was used in experiments where they were attached to *P*. *aeruginosa* biofilms during initiation (Fig 4E and 4F), the clusters are less compact as compared to those formed by strain 2868 and 2871.

## Discussion

Due to the high incidence of human *Campylobacter* infections worldwide it is important to understand the ability of the organism to persist in the environment and the food chain. Since biofilm formation has been suggested to play a significant role in protecting *C*. *jejuni* cells from adverse conditions [12, 13], a clearer understanding on how these bacteria behave under conditions that mimic the conditions they are likely to face in the environment may serve as a framework for development of new strategies to prevent and control *C*. *jejuni*.

In the present study, attachment of two *C*. *jejuni* strains isolated from poultry and *C*. *jejuni* ATCC 33291 under flow and aerobic conditions on different abiotic surfaces was investigated. All the three *C*. *jejuni* strains tested were found to be able to attach to different surfaces tested to different degrees. This suggested that attachment by *C*. *jejuni* differs significantly between strains, as highlighted in a literature review on *C*. *jejuni* biofilms [18].

The nature of the surface has been suggested to affect the extent of attachment. From the results obtained, one of the *C*. *jejuni* strains tested (*C*. *jejuni* strain 2871) attached significantly more to more hydrophobic surfaces (plastic) as compared to stainless steel. This is consistent with previous studies which showed that bacteria attach better to hydrophobic surface as compared to hydrophilic surfaces due to higher hydrophobic interaction between the hydrophobic components on bacterial surfaces and the substratum [27–30].

Attachment by all the strains regardless of other treatments was significantly lower under flow conditions as compared to static conditions. It is noteworthy that while 2 log of cells were recovered under static conditions in the mono-species experiments for strain 2868, this strain was unable to attach under flow conditions. These results are consistent with previous studies that reported that *C*. *jejuni* strains which attached strongly to glass in static culture failed to attach to glass or any other surfaces when they were grown under shaking conditions (80-100rpm) [14]. These authors also reported that *C*. *jejuni* failed to form biofilms in a Robbins device with a flow rate of 300ml h^-1^, a rate which was widely used in other studies, [31] or even at a lower flow rate of 10ml h^-1^ [14]. Studies carried out by Ica, et al. [20] also reported that mono-species *C*. *jejuni* biofilms were unable to persist at a flow rates of 1 to 2.5 ml/min [20]. The attached *C*. *jejuni* in our study, regardless of whether they were present alone or with *P*. *aeruginosa*, were considered fragile as they were disrupted under the flow conditions used as seen in the SEM images (Figs 2–4). Association with more compact biofilms connected by extensive EPM might provide better attachment and allow *C*. *jejuni* to persist better under static conditions as compared to flow conditions. These findings suggest that it is unlikely that *C*. *jejuni* survives in watering supplies and plumbing systems of animal processing plants as a single species either attached or in biofilm.

*C*. *jejuni* cells attached to abiotic surfaces appeared to be cocci in shape when incubated under aerobic conditions at 25°C (Fig 5) indicating that the cells were stressed under the conditions they are likely to face in the environment. Previous studies have proposed that the synergistic interactions between *C*. *jejuni* and other bacterial species in the environment might enhance the survival of *C*. *jejuni* outside the hosts in external environment [19, 20]. The results from the current study showed that for both of the *C*. *jejuni* strains isolated from poultry, higher number of cells were recovered when *C*. *jejuni* was co-cultured with *P*. *aeruginosa* as compared to alone under both static and flow conditions. SEM images (Figs 2 to 3) demonstrated that under static conditions in the presence of *P*. *aeruginosa* biofilms *C*. *jejuni* appeared as clusters of cells surrounded with extensive of EPM. This is very different from the *C*. *jejuni* cells present on pre-existing *P*. *aeruginosa* biofilm, which appeared to be a single layer of cells connected with EPM. EPM has been reported to be an essential component of the bacterial biofilm and dependent on bacterial species and isolate [32]. There is, however, relatively little information available on the structure and composition of EPM produced by *C*. *jejuni* [33]. The clusters of cells and the extensive EPM that formed in mixed-species biofilm might play a role in capturing *C*. *jejuni* and provide protection to them, allowing them to survive better even under flow conditions as compared to alone. Other studies have shown the presence of a higher level of extracellular polymeric substances with a more diversified chemical composition in dual-species biofilm of *C*. *jejuni* and *Staphylococcus aureus*, *Salmonella enterica*, or *Pseudomonas aeruginosa*, as compared to mono-species *C*. *jejuni* biofilm [34]. A previous study has suggested that in a mixed species biofilm, the presence of a bacterial species that produces EPM may lead to the integration of other species that do not synthesize it [32]. In the current study it is likely that the *P*. *aeruginosa* biofilm is providing the EPM which enhances *C*. *jejuni* attachment.

The two *C*. *jejuni* strains isolated from poultry attached better when co-cultured with *P*. *aeruginosa* which may also provide better protection for them under atmospheric conditions as well as under flow conditions as suggested by previous studies [20, 21]. Numbers of *C*. *jejuni* cells recovered from pre-existing *P*. *aeruginosa* biofilms was significantly higher as compared to that in the mono-species experiments under specific conditions (static conditions for strain 2865 and flow conditions for strain 2871) (Fig 1). When *C*. *jejuni* 2868 was added to pre-existing *P*. *aeruginosa* biofilms, no clustering of cells were observed (Fig 2A and 2B) but a single layer of cells that formed small aggregates with EPM was apparent. *C*. *jejuni* may become entrapped within these aggregates, enhancing their attachment and reducing stress under normal atmospheric conditions as compared to their attachment to abiotic surfaces. When *C*. *jejuni* 2871 was added to pre-existing *P*. *aeruginosa* biofilms a single layer of bacterial cells connected with flattened EPM (Fig 3A and 3B) was apparent. This may be the reason why no significant differences in the number of *C*. *jejuni* cells recovered from pre-existing *P*. *aeruginosa* biofilm as compared to those in the mon-species experiments under static conditions for strain 2871 was observed (Fig 1). For *C*. *jejuni* ATCC 33291, there was no significant difference in the number of *C*. *jejuni* cells recovered from *P*. *aeruginosa* biofilms as compared to those in the mon-species experiments under both static and flow conditions (Fig 1). SEM images also demonstrated that clusters of cells formed when *C*. *jejuni* ATCC 33291 were attached to *P*. *aeruginosa* biofilms during initiation (Fig 4E and 4F) are less compact as compared to those formed by strains 2868 and 2871. This suggested that ATCC 33291 does not coexist well with *P*. *aeruginosa* to form a stronger biofilm as compared to attachment alone. Similar results was obtained in a previous study which showed that *C*. *jejuni* was unable to coexist in most of the mixed-species biofilms that contained *P*. *aeruginosa* [15]. The difference in the results observed might be due to strain variation.

The results of this study showed uniquely that *C*. *jejuni* attachment is enhanced by biofilms of *P*. *aeruginosa*, particularly if cells are present during biofilm initiation, as compared to mono-species experiments when incubated under atmospheric and ambient temperature conditions as well as under flow conditions. This suggests that attachment to biofilms of other bacteria species may provide protections to *C*. *jejuni* against oxygen stress and shear forces which then contribute to their persistence outside the host in the environment. Further studies to investigate attachment and survival of *C*. *jejuni* under conditions relevant to the environment these bacteria are likely to face, rather than under more favourable laboratory conditions of atmosphere, temperature and low-sheer are required. This will provide a better understanding on how *C*. *jejuni* interact with the environment and what potential this has to enhance their persistence under conditions which are detrimental to them. This study supports the hypothesis that *C*. *jejuni* do not form biofilms *per se* under conditions they encounter in the environment but simply attach to surfaces or biofilms of other species.

## Supporting information

S1 Table Multi-factor ANOVA showing the interaction of attachment with specific factor(s): Bacterial Strain; Type of Abiotic Surfaces, Static or Flow Conditions and Type of Attachment. (DOCX)
