# Supplementary material for: Association of some Campylobacter jejuni with Pseudomonas aeruginosa biofilms increases attachment under conditions mimicking those in the environment
Source: PLoS One. 2019 Apr 10;14(4):e0215275. doi: 10.1371/journal.pone.0215275 (PMC6457560; doi:10.1371/journal.pone.0215275)
Supplement: S1 Table — (DOCX) [file pone.0215275.s001.docx]

S1 Table. Multi-factor ANOVA showing the interaction of attachment with specific factor(s): Bacterial Strain; Type of Abiotic Surfaces, Static or Flowing Condition and Type of Attachment.

| Tests of Between-Subjects Effects | | | |  |
| --- | --- | --- | --- | --- |
| Dependent Variable: Cell Count | | | |  |
| Source | df | F | Sig. | |
| Corrected Model | 53 | 13.486 | .000 | |
| Intercept | 1 | 3241.322 | .000 | |
| Bacterial Strains | 2 | 149.360 | .000 | |
| Surface | 2 | 3.140 | .047 | |
| Static/ Flowing | 1 | 213.726 | .000 | |
| Attachment | 2 | 49.039 | .000 | |
| Bacterial Strains * Surface | 4 | .349 | .844 | |
| Bacterial Strains * Static/Flowing | 2 | 14.762 | .000 | |
| Bacterial Strains * Attachment | 4 | 8.663 | .000 | |
| Surface * Static/Flowing | 2 | .412 | .663 | |
| Surface * Attachment | 4 | .148 | .963 | |
| Static/Flowing * Attachment | 2 | .116 | .890 | |
| Bacterial Strains * Surface * Static/Flowing | 4 | .267 | .898 | |
| Bacterial Strains * Surface * Attachment | 8 | .564 | .805 | |
| Bacterial Strains * Static/Flowing * Attachment | 4 | 5.546 | .000 | |
| Surface * Static/Flowing * Attachment | 4 | .317 | .866 | |
| Bacterial Strains * Surface * Static/Flowing * Attachment | 8 | .215 | .988 | |
| Error | 108 |  |  | |
| Total | 162 |  |  | |
| Corrected Total | 161 |  |  | |
| a. R Squared = .869 (Adjusted R Squared = .804) | | | |  |
